# Comparative Analysis of Chemotherapy Resistance Mechanisms in Humans and Companion Animals

**DOI:** 10.3390/vetsci12080747

**Published:** 2025-08-12

**Authors:** Daniel Luiz de Miranda Cravo, Pedro Antônio Bronhara Pimentel, Ana Paula Vargas Garcia, André Luiz de Moura Junqueira, Fabiana Sanches Soares, Antonio Giuliano, Angel Almendros, Rodrigo dos Santos Horta

**Affiliations:** 1 Department of Veterinary Medicine and Surgery, Veterinary School, Universidade Federal de Minas Gerais, Belo Horizonte 31270-901, Brazil; danielcravo@ufmg.br (D.L.d.M.C.); pedrobpimentel@ufmg.br (P.A.B.P.); almj7657@ufmg.br (A.L.d.M.J.); fabiisanches@ufmg.br (F.S.S.); 2 Department of Pathology, Institute of Biology, Universidade Federal de Minas Gerais, Belo Horizonte 31270-901, Brazil; 3 Harvest Veterinary Oncology Center, Kwai Fung, Kwai Chung, Kowloon, Hong Kong 999077, China; agiulian@cityu.edu.hk; 4 Department of Veterinary Clinical Sciences, Jockey Club College of Veterinary Medicine, City University of Hong Kong, Kowloon, Hong Kong 999077, China

**Keywords:** neoplasm, mutations, chemotherapy, dogs, cats

## Abstract

The transition from a normal to a cancerous cell involves a multistep process driven by genetic alterations. Malignant cells progressively acquire traits that enhance their survival within the host, including the ability to evade apoptosis, stimulate neovascularization, and escape immune surveillance. These adaptations allow cancer to grow uncontrollably, invade adjacent tissues, and metastasize to distant organs, ultimately impairing normal physiological functions and complicating treatment efforts. Among the most significant clinical challenges is chemotherapy resistance, which diminishes the effectiveness of conventional therapies and contributes to disease progression. Addressing this issue requires integrated and multidisciplinary approaches. This review investigates and compares the mechanisms of chemotherapy resistance identified in humans and companion animals. By examining both shared and species-specific features, this analysis aims to support the development of more effective and translational treatment strategies.

## Simple Summary

Cancer is a complex disease that affects not only humans but also animals, such as dogs and cats. One of the main challenges in its treatment is the development of resistance to chemotherapy, which progressively reduces the effectiveness of therapeutic approaches. This review provides a comparative analysis of how chemotherapy resistance arises in humans and in dogs and cats with spontaneous tumors. It describes the various strategies employed by cancer cells to evade drug-induced cell death, including drug efflux, enhanced DNA repair mechanisms, metabolic reprogramming, and inhibition of programmed cell death. Resistance may be intrinsic—present before treatment—or acquired during therapy. Many of these resistance mechanisms are conserved across species. Understanding these shared pathways may guide the development of more effective therapies, ultimately improving treatment outcomes and quality of life in both human and veterinary oncology.

## 1. Introduction

Cancer is a disorder driven by the accumulation of genetic and epigenetic alterations that reshape the genome, ultimately transforming normal cells into malignant ones [1]. Neoplastic cells progressively acquire a set of traits known as the hallmarks of cancer. These include the ability to sustain proliferative signaling, evade growth suppressors, resist cell death and apoptosis, achieve replicative immortality, induce and access blood vessel formation, and promote inflammation. Additional hallmarks include genomic instability, the presence of senescent cells, polymorphism, and active manipulation of the microbiome to promote tumor progression by influencing local immunity and metabolism. Cancer cells also exhibit nonmutational epigenetic reprogramming, phenotypic plasticity, reprogramming of cellular metabolism, immune evasion, and the capacity to metastasize [2,3,4]. Humans and companion animals develop similar spontaneous tumors (e.g., osteosarcoma, hemangiosarcoma, lymphoma, and leukemia), soand many epidemiological features are shared across species [5,6]. Although the mechanisms driving tumor development and progression may vary, they often share common features. These include evasion of cell death and uncontrolled proliferation, which point to partially conserved biological pathways [6].

The genomic instability of neoplastic cells, combined with the breakdown of proliferation control mechanisms, drives the striking heterogeneity observed within tumors. This variability poses a significant challenge to cancer treatment, as distinct subclones of cells may exhibit divergent responses to the same therapy [7]. Multidrug regimens and combined chemotherapy protocols may apply selective pressure, reducing susceptible cell populations while favoring the survival of some resistant clones [8]. Tumor progression, metastasis, and therapy resistance are further fueled by acquired genetic alterations, clonal evolution, and dynamic interactions within the tumor microenvironment. These factors play a critical role in cancer evolution and its capacity to evade and resist therapies [2].

Chemotherapeutic agents are categorized into five classes based on shared mechanisms of action and resistance. The main classes are alkylating agents, antimetabolites, antitumor antibiotics, topoisomerase inhibitors, and tubulin-binding drugs [9]. Exceptions include platinum-derivatives, which act in a very similar way to alkylating agents; hydroxyurea, which acts similarly to antimetabolites; and L-asparaginase, which uniquely disrupts protein synthesis by depleting asparagine during the G1 phase. Beyond conventional chemotherapy, modern cancer treatment increasingly utilizes immunotherapies, including immune checkpoint inhibitors like anti-PD-1/PD-L1 therapies, which aim to reactivate the host’s immune system against tumor cells. While these strategies have revolutionized oncology, the development of resistance mechanisms remains a significant clinical challenge also for these novel approaches [9]. Chemotherapy resistance is a major reason for therapeutic failure [10]. Resistance mechanisms can be classified into intrinsic (primary) or acquired. Intrinsic resistance is related to pre-existing cellular traits that render cancer cells inherently unresponsive to a chemotherapeutic drug [10,11]. Acquired resistance is related to mechanisms developed over time as cancer cells adapt to chemotherapy through mechanisms like epigenetic changes or clonal selection of resistant subpopulations [11]. Extrinsic resistance, distinct from cellular mechanisms, arises from factors in the tumor microenvironment and is associated with limited drug efficacy. This can occur through two main mechanisms, poor drug delivery or insufficient prodrug activation. Poor drug delivery may occur due to anatomical barriers (e.g., blood–brain or blood–ocular barriers) or tumor structural features, like avascular regions, fibrotic stroma, stiff extracellular matrix, or elevated interstitial pressure. Insufficient prodrug activation may occur with prodrugs like cyclophosphamide, which needs to be activated by liver enzymes, therefore requiring functional liver integrity [9,10,11].

In veterinary oncology, the study of chemotherapy resistance mechanisms is still developing, especially when compared to the more comprehensively explored pathways in human medicine. Nonetheless, recent comparative studies have begun to shed light on these mechanisms across species. Dogs and cats are considered valuable translational models in oncology due to their spontaneous tumor development, genetic similarities with humans, comparable environmental exposures, and similar responses to treatment. For example, mammary gland tumors are the most common neoplasms in both unspayed female dogs and women. In human breast cancer, the overexpression of drug-efflux transporters such as P-glycoprotein (PgP) has been linked to resistance to chemotherapy. A similar phenomenon has been observed in canine mammary tumors, where altered gene expression may activate comparable efflux systems, contributing to therapeutic resistance [12].

Species-specific differences in chemotherapy metabolism play a crucial role in treatment efficacy and toxicity, particularly due to variations in hepatic enzyme activity. The liver is the primary site for drug biotransformation, and its enzymatic profile differs significantly between humans, dogs, and cats [13]. For instance, cytochrome P450 (CYP) isoenzymes, which are central to phase I metabolism, exhibit distinct patterns of expression and activity across species, influencing the rate and extent of drug activation or detoxification [14,15]. Cats have a limited capacity for glucuronidation due to reduced expression of certain UDP-glucuronosyltransferase (UGT) enzymes, which can lead to slower clearance and increased toxicity of some chemotherapeutic agents [15]. These interspecies metabolic differences must be carefully considered when extrapolating treatment protocols or interpreting comparative oncology data, as they can significantly impact drug bioavailability, efficacy, and adverse-effect profiles.

This literature review synthesizes recent advances in understanding tumor resistance mechanisms, offering a comparative analysis of conserved and divergent pathways between humans and companion animals. By bridging insights from veterinary and human oncology, this work aims to identify translational opportunities for overcoming therapeutic resistance in both species.

## 2. Materials and Methods

Our search strategy aimed to locate published studies written in English or Portuguese. An initial limited search was conducted via PubMed to identify articles on chemotherapy resistance mechanisms in humans, dogs, and cats, and to determine relevant mesh terms and keywords. The text words contained in titles, abstracts, and index terms were then used to develop a comprehensive search strategy, which was applied across the main platforms: Web of Science, CAPES, and PubMed. Key concepts included “chemotherapy resistance,” “drug resistance,” “cancer,” “tumor,” “human,” “dog,” “canine,” “cat,” and “feline.” Reference lists of all studies selected for critical appraisal were screened for additional relevant publications. The databases were searched between May 2024 and January 2025. No date restrictions were applied.

## 3. Major Tumoral Resistance Mechanisms

**Figure 1:**
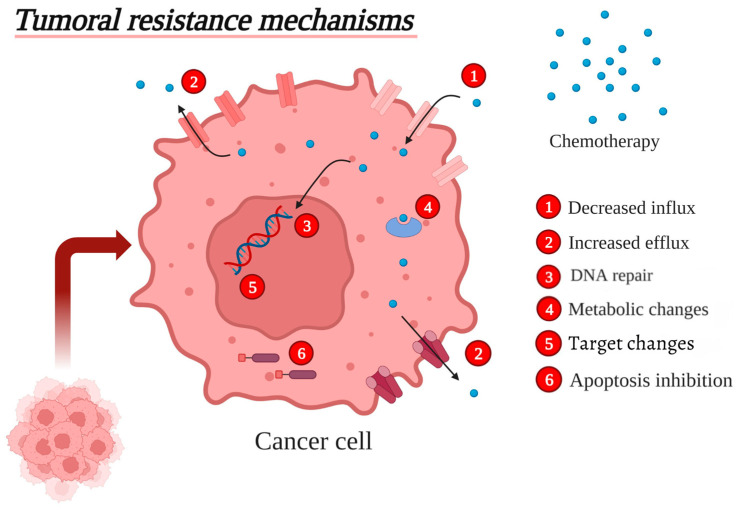
Schematic representation of a neoplastic cell representing six key mechanisms of resistance to chemotherapeutic agents: (1) decreased drug influx, (2) increased drug efflux, (3) DNA repair activation, (4) metabolic adaptations, (5) target modifications, and (6) inhibition of apoptosis. Figure created in 2023 version of Biorender^®^.

Chemoresistance in tumors arises through intrinsic (primary) or acquired mechanisms which enable the neoplasm survival [10]. Shared mechanisms underscore conserved pathways in cancer progression across species. For example, in canine lymphoma, intrinsic resistance mechanisms have been reported in patients who fail to achieve complete remission with initial chemotherapy protocols, while acquired resistance is commonly observed in relapsed cases after prior treatment exposure [15,16]. Historically, resistance was attributed to the selection of pre-existing resistant subclones during therapy, which was considered the main cause of treatment failure [17]. However, emerging evidence underscores that the mechanisms behind resistance are more complex than initially thought [17]. As illustrated in Figure 1, resistance mechanisms in neoplastic cells include (1) decreased influx of chemotherapeutic drugs, (2) increased efflux of chemotherapeutic drugs, (3) DNA repair, (4) metabolic changes, (5) target changes, and (6) apoptosis inhibition.

Resistance mechanisms are commonly activated in cancers that exhibit poor treatment responsiveness [2]. Emerging evidence highlights that the expression of markers related to pluripotency is crucial for tumor survival and resistance to treatment [2]. Pluripotency refers to the ability of undifferentiated cancer stem cells to differentiate into diverse tumor cell populations [17]. However, despite this inherent pluripotency, the cancer cells tend to retain a less differentiated phenotype to preserve their high proliferation potential. These cells multiply faster and can lead to the regeneration of the tumor mass even after treatment, enabling resistance mechanisms [2].

### 3.1. Decreased Influx of Chemotherapeutic Drugs

The intracellular concentration of chemotherapeutic agents depends on their transport mechanisms, active transport, passive transport, or facilitated diffusion [11]. Effective drug action requires sufficient influx to achieve a cytotoxic concentration gradient within tumor cells [9,11]. Resistance can arise from reduced expression or dysfunction of membrane transporters responsible for drug uptake, thereby lowering intracellular drug levels [18,19].

The balance between the efflux and influx of chemotherapeutic agents depends on the activity of membrane transporter proteins. Reduced influx of anticancer drugs can result from either a reduction in the binding affinity of the drug and its transporter or a decreased transporter expression.

Different chemotherapeutic groups utilize distinct membrane transporters for cellular entry. Platinum-based drugs (e.g., cisplatin and carboplatin) utilize copper transporter CTR1 [20]. Topoisomerase inhibitors, such as irinotecan and topotecan, as well as anthracyclines, are actively transported into the cell by organic anion-transporting polypeptides [21,22]. Antimetabolites enter the cell via nucleoside transporters [23]. Among alkylating agents, streptozotocin enters cells via glucose transporters, like GLUT2 [24]. Lipophilic drugs (e.g., cyclophosphamide and doxorubicin) passively diffuse across membranes or use protein-facilitated transport [25,26].

Decreased influx of chemotherapeutic drugs represents a significant mechanism of chemoresistance in human cancers, leading to reduced intracellular concentrations of drugs. This primarily occurs through the downregulation or expression of less functional variants of plasma membrane transporters, particularly those belonging to the Solute Carrier Family (SLC) [27]. For instance, reduced expression or functionality of the plasma membrane transporters proteins OATP1B1 and OATP1B3 (encoded by SLCO1B1 and SLCO1B3 genes) has been linked to lower accumulation of antitumor drugs in human liver cancer, including hepatocellular carcinoma (HCC) and cholangiocarcinoma (CGC) [27]. Additionally, alterations in CTR1 (SLC31A1), a copper transporter that also transports platinum drugs, affect platinum-drug sensitivity in cancer chemotherapy [27]. Similarly, decreased expression of the plasma membrane transporter OCT1 (SLC22A1 gene) is a common feature in primary liver cancers and colon cancer, affecting the uptake of drugs like sorafenib [28]. In summary, impaired drug uptake due to dysfunctional membrane transporters—especially within the SLC family—plays a key role in chemoresistance [27,28]. Understanding these mechanisms is essential to improve drug delivery and therapeutic outcomes in cancer treatment.

In companion animals, decreased influx of chemotherapeutic agents is a well-established mechanism of drug resistance in veterinary oncology. Reduction of intracellular drug accumulation, due to altered expression or function of membrane transporters, plays a significant role in chemoresistance in canine lymphoma [29]. Transporters such as P-glycoprotein, MRP1, and BCRP are implicated, and while most research has focused on efflux, impaired influx mechanisms also contribute to subtherapeutic drug levels [29]. Additionally, although studies in felines are more limited, emerging data suggest that functional variations in transporters like ABCG2 may influence drug pharmacokinetics and resistance patterns in feline tumors, and differences in the activity of the feline ABCG2 transporter may reduce the efflux of platinum compounds from cancer cells, potentially altering the drug’s intracellular concentration and impacting both its effectiveness and the development of resistance [30]. These findings emphasize the importance of understanding drug transporter function across species to optimize chemotherapy efficacy in veterinary patients.

Understanding these transport mechanisms is crucial to treatment efficacy, as targeting transporters expressed in specific tissues can enhance drug selectivity, improve therapeutic outcomes, and mitigate resistance [24].

### 3.2. Increased Efflux of Chemotherapeutic Drugs

Drug efflux of chemotherapeutic drugs is also related to resistance, directly reducing drug concentrations. This process is mediated by membrane-bound transporter proteins, which transfer the drugs from the intracellular to the extracellular environment [11]. Among these, the ATP-binding cassette (ABC) transporter family is an important group in expelling diverse anticancer drugs, such as the anthracyclines, topoisomerase inhibitors, antimetabolites, platinum compounds, and microtubule inhibitors [11,31,32,33]. Structurally, ABC transporters consist of membrane-spanning domains and nucleotide-binding domains that enable the passage of the substrate in a selective and energy-dependent way by the hydrolysis of ATP [34,35].

Among the ABC transporters, the multidrug resistance protein 1 (MDR1, P-glycoprotein, and ABCB1), the MDR-associated protein 1 (MRP1 and ABCC1), the breast cancer resistance protein (BCRP and ABCG2), and the MDR-associated protein 3 (MRP3 and ABCC3) are the most important and well-characterized mediators related to multidrug resistance in humans and animals [33,34,36]. These proteins selectively export lipophilic chemotherapy drugs, including folate analogs; microtubule inhibitors drugs; and targeted therapies [11,35].

For instance, methotrexate resistance involves reduced influx due to impaired function of the reduced folate carrier 1 (RFC-1) and increased efflux via MRP3 overexpression [18]. Additionally, reduction in the activity of the enzyme folylpolyglutamate synthetase (FPGS) or increased activity of γ-glutamyl-hydrolase (GGH) activity inhibits methotrexate polyglutamation (glutamate addition), reducing intracellular drug retention and efficacy [18].

In humans, the increased efflux is a major mechanism of resistance. This process is primarily mediated by the ABC proteins, which actively pump the drug out of cells using energy from ATP hydrolysis [37]. The main ABC transporters involved in this multidrug resistance include P-glycoprotein (MDR1), multidrug resistance-associated proteins (MRP1), and breast cancer resistance protein (BCRP) [38,39]. They efficiently export a broad range of lipophilic drugs, such as anthracyclines, topoisomerase inhibitors, antimetabolites, and platinum compounds [39]. This type of resistance happens in several cancers in humans, like acute myeloid leukemia, breast cancer, colorectal cancers, and small-cell lung cancer [37,40].

In veterinary oncology, increased drug efflux is also recognized as a key mechanism of resistance. Similar to humans, this process is mainly mediated by ABC transporters, such as P-glycoprotein (ABCB1) and multidrug resistance-associated proteins [41]. In dogs, overexpression of ABCB1 has been documented in certain lymphomas and osteosarcomas, contributing to resistance against drugs like doxorubicin and vincristine [16,41]. Although studies in cats are more limited, emerging evidence suggests that transporters such as ABCG2 may also play a role in this context, increasing chemotherapy resistance in feline low-grade alimentary lymphoma, for example [42]. These efflux mechanisms present a major challenge in veterinary oncology.

#### 3.2.1. Multidrug Resistance Protein 1

Multidrug resistance protein 1 (MDR1/P-glycoprotein) is a protein encoded by the ABCB1 gene, classified as a phosphorylated and glycosylated protein with 1280 amino acids. It is responsible for transport of neutral or positively charged hydrophobic and amphipathic substances, contributing to cellular protection against potentially toxic xenobiotics [33,42,43].

Physiologically, MDR1 is highly expressed in epithelial cells of excretory organs (e.g., pancreatic and bile ducts cells) and endothelial cells of the brain–blood barrier [44]. In cancer cells, the expression of MDR1 is more commonly observed and can be further induced by exposure to chemotherapeutic agents, including glucocorticoids, in both humans and dogs [10,41]. MDR1 actively exports diverse chemotherapeutics, such as tubulin-binding drugs like taxanes (paclitaxel and docetaxel), vinca alkaloids (vincristine, vinblastine, and vinorelbine), and anthracyclines (doxorubicin) [33,36,44,45].

#### 3.2.2. MDR-Associated Protein 1

Multidrug resistance-associated protein 1 (MRP1), encoded by the ABCC1 gene, is another ABC-transporter protein linked to chemoresistance [31,33,46,47]. It exports a broad range of substrates, including heavy-metal anions; toxicants; and conjugates of glutathione, glucuronide, and sulfate, across tissues such as cardiac and skeletal muscles [33,46,47]. Structurally, MRP1 differs from MDR1 by possessing an additional transmembrane domain and exhibits substrate specificity for conjugated compounds (e.g., glutathione-bound metabolites) [33,48,49].

In human oncology, the overexpression of MRP1 has been extensively linked to resistance to chemotherapy in malignancies such as acute lymphoblastic leukemia and non-small-cell lung carcinoma [47,49]. This upregulation drives resistance to various anticancer drugs, including anthracyclines (doxorubicin), topoisomerase inhibitors (camptothecins), antimetabolites, epipodophyllotoxins, and vinca alkaloids [33,48,49].

#### 3.2.3. Breast Cancer Resistance Protein

Breast cancer resistance protein (BCRP), encoded by the ABCG2 gene, is a key contributor to chemotherapy resistance [33,50]. This transporter has 655 amino acids, consisting of six transmembrane helices and one single ATP-binding site [51]. It is expressed in diverse tissue, including capillary endothelial cells and the pancreas, where it plays a protective role in homeostasis by excreting xenobiotics and toxic compounds [51,52].

The overexpression of this transporter correlates with resistance to anticancer drugs, such as canine mammary gland tumor, human acute myeloid leukemia, human colon carcinoma, human breast carcinoma, human gastric carcinoma, human fibrosarcoma, human non-small-cell lung cancer, human glioblastoma and human myeloma, human ovarian cancer cell, and human prostate cancer cell [33,51,52]. BCRP functions as a homodimeric/oligomeric efflux pump associated with resistance to anthracyclines (doxorubicin and epirubicin), anthracenes (bisantrene and mitoxantrone), topoisomerase inhibitors (camptothecin derivates and topotecan), flavopiridol, antimetabolites (methotrexate), imatinib, gefitinib, and nilotinib [33,51,52,53,54]. In cats, a polymorphism in the ABCG2 gene results in non-functional or less efficient BCRP proteins [55]. While this polymorphism may reduce resistance to certain drugs, it increases the risk of adverse events, though other resistance mechanisms may persist [55].

#### 3.2.4. Increased ABC Transporters

Increased expression of ABC transporters enhances drug efflux, as these proteins actively expel compounds from the intracellular environment to the extracellular environment [36]. In tumor cells, overexpression of these transporters reduces intracellular chemotherapeutic concentrations, diminishing treatment efficacy and driving resistance [34].

MDR1 and MRP1 are responsible for the efflux of vinblastine, contributing to resistance [12]. The efflux of cisplatin is mediated by MDR1, BCRP, and MRP1, while cyclophosphamide is mediated by BCRP [12]. Resistance to doxorubicin and vincristine in canine lymphoma is completely reversed using an MDR1 inhibitor, underscoring the clinical relevance of targeting efflux mechanisms [16]. Elevated expression of the ABC transporters is associated with poorer treatment outcomes due to enhanced drug efflux, thereby reducing the treatment effectiveness [16].

### 3.3. Enhanced DNA Repair

DNA repair is a widely recognized mechanism of drug resistance oncology [35,56]. Chemotherapy agents such as alkylating agents, platinum-based drugs, and topoisomerase inhibitors can cause direct or indirect DNA damage that can result in tumor-cell apoptosis [35]. While DNA repair is a physiological process that safeguards genomic integrity, it also serves as a neoplastic mechanism of resistance, as the tumor cell manages to repair DNA damage caused by chemotherapy [57]. Resistance to these agents occurs due to the DNA repair systems present in cancer cells, including the nucleotide excision repair system (NER) and homologous recombination repair (MRR) mechanisms [32,56].

Cisplatin, a platinum-based chemotherapeutic, exerts cytotoxicity by forming DNA crosslinks that disrupt replication [56]. However, the tumor cells execute several repair mechanisms to avoid the lesions caused by the chemotherapy drug. These repair mechanisms involve different strategies to avoid cell damage, like DNA repair and replication of a damaged molecule [17].

Despite these mechanisms, resistance to cisplatin in humans often involves additional mechanisms, including reduced drug influx, increased efflux via ABC transporters, and detoxification pathways that neutralize the drug’s effects [58]. One of the most prominent pathways involved is the nucleotide excision repair (NER), through the action of the ERCC1-XPF endonuclease complex, responsible for removing cisplatin-induced DNA adducts [58]. Elevated expression of the ERCC1 has been associated with reduced cisplatin sensitivity in non-small-cell lung cancer and ovarian cancer [59]. The upregulation or reactivation of these DNA repair mechanisms allows cancer cels to survive and proliferate despite chemotherapeutic damage [58].

In veterinary oncology, DNA repair mechanisms have also been implicated in chemoresistance in both canine and feline tumors, although the available data are more limited compared to human studies [60]. Platinum-based drugs, like cisplatin and carboplatin, are commonly used in companion animal medicine and function by forming DNA crosslinks that impar replication and inducing apoptosis [61]. However, canine and feline cancer cells can use DNA repair pathways, such as NER (like humans), and homologous recombination (HR) to counteract this damage [60]. Canine osteosarcoma and lymphoma can have an overexpression of DNA repair genes, contributing to reduced sensitivity to DNA-damaging drugs [29,60]. In felines tumors, the mechanisms are not very explained; however, a comparable mechanism with canine and humans may likely occur [61].

### 3.4. Metabolic Changes

Cancer is characterized by metabolic dysregulation resulting from the disruption of cellular homeostasis, leading to uncontrolled growth and impaired cellular functions, which can invade adjacent tissues or metastasize [56,62]. Cancer cells need to reprogram their catabolic and anabolic processes, thereby adjusting their energy metabolism and producing a large amount of energy and biomass needed to sustain rapid proliferation, invasion, and metastasis, and therefore survive in a hostile microenvironment [63]. This metabolic rewiring is orchestrated by oncogenes and tumor-suppressor genes that directly regulate key pathways, collectively altering cellular metabolism to fuel unchecked division [64,65].

A critical mechanism of chemotherapy resistance involves metabolic adaptation. Cancer cells dynamically adjust their metabolism to counteract drug effects—for example, by enhancing adaptive capacity under chemotherapeutic stress, thereby reducing treatment efficacy [35,66,67]. Such metabolic flexibility, including the rewiring of central carbon and nucleotide pathways, allows cells to evade drug toxicity and persist [58,65]. Additionally, cellular dormancy, a non-proliferative state, leads to insusceptibility to chemotherapy, which can be reversed when the circumstances favor their proliferation [68].

Tumor cells further adapt by modulating oxidative metabolism, toggling between aerobic glycolysis and oxidative phosphorylation in response to microenvironmental cues, and thus promoting resistance [69]. The cancer cells also have impaired mitochondrial respiratory chain, which also contributes to the metabolic changes [69].

Enzymes play a pivotal role by modulating the intracellular and extracellular concentrations of chemotherapeutic agents. Phase I reactions (e.g., oxidation, reduction, and hydrolysis) and phase II reactions (e.g., conjugation and acetylation) are critical for neutralizing toxins in normal cells [55]. Once the drugs enter the cells, detoxification pathways rapidly inactivate chemotherapeutics.

Glutathione S-transferases (GSTs) are a family of phase II detoxification enzymes that catalyze the conjugation of several compounds to glutathione, resulting in its inactivation [32]. The upregulation of the glutathione S-transferase isozyme enhances detoxification of chemotherapeutic agents in solid neoplasms through chemical conjugation reactions, reducing drug efficacy and worsening clinical outcomes [70].

The enzyme thymidylate synthase (TS) and its coding gene (TYMS) are prognostic markers of the effectiveness of treatment with antifolates for several malignancies in humans, such as colorectal cancer [71]. TS confers resistance by converting antifolates into inactive metabolites, blunting their antitumor effects [71]. Gemcitabine hydrochloride, an antimetabolite used for pancreatic adenocarcinoma in humans, requires phosphorylation in the intracellular environment by the enzyme deoxycytidine kinase, encoded by the DCK gene [71]. Resistance arises from DCK deficiency, which limits gemcitabine activation and abolishes its therapeutic impact—a mechanism inversely related to GST- or TS-mediated resistance [71].

In humans, tumor cells modify core metabolic pathways to sustain rapid proliferation [72]. Under normal circumstances, metabolic pathways are tightly regulated to ensure cellular homeostasis [72]. However, in response to disease, particularly cancer, the metabolic landscape is drastically reprogrammed [72]. The tumor cells often shift from oxidative phosphorylation to aerobic glycolysis (this is called Warburg effect), enabling rapid ATP production and biomass synthesis to support uncontrolled proliferation [73]. Additionally, chronic conditions such as diabetes mellitus, obesity, and neurodegenerative diseases are associated with systemic metabolic dysregulation, including insulin resistance, altered lipid handling, and mitochondrial dysfunction [74]. These disruptions contribute not only to disease progression but also to decreased therapeutic responsiveness [74]. In inflammatory or hypoxic environments, human cells further adapt their metabolic behavior to ensure survival, modifying glucose, amino acid, and fatty acid metabolism [74]. Several human tumors exhibit distinct metabolic phenotypes that support their aggressiveness and resistance to therapy. For example, glioblastoma multiforme, an aggressive brain tumor, is known for its strong dependence on aerobic glycolysis and elevated glutamine metabolism, supporting rapid growth in a hypoxic environment [75]. Pancreatic ductal adenocarcinoma displays an extensively reprogrammed metabolism, relying on autophagy and macropinocytosis to fuel anabolic pathways, while also showing resistance to oxidative stress [76]. In colorectal cancer, alterations in the tricarboxylic acid (TCA) cycle and overexpression of lactate dehydrogenase A (LDH-A) contribute to lactate accumulation and tumor progression [77]. These examples illustrate how tumor-specific metabolic traits not only enable survival under stress conditions but also represent promising targets for therapeutic intervention.

In dogs, as in humans, tumor cells undergo significant metabolic reprogramming to support rapid proliferation, survival in hostile microenvironments, and resistance to therapy [78]. One of the best-documented examples is canine lymphoma, where neoplastic lymphocytes show increased glycolytic activity and upregulation of glucose transporters (e.g., GLUT1), reflecting a Warburg-like effect [78]. Canine mammary gland tumors often demonstrate dysregulated lipid metabolism, with elevated expression of enzymes involved in fatty acid synthesis and β-oxidation, supporting membrane biosynthesis and energy production [79]. In oral malignant melanomas, an aggressive neoplasm in dogs, metabolic adaptations include enhanced mitochondrial activity and resistance to oxidative stress, contributing to their high metastatic potential [80]. These findings suggest that, similar to human cancers, canine tumors exploit specific metabolic pathways not only for growth and invasion but also for drug resistance.

#### Increased Drug Inactivation and Reduced Activation of Chemotherapeutic Agents

The pharmacokinetic behavior of a chemotherapeutic agent profoundly influences treatment efficacy and is intricately linked to chemoresistance [11]. For a drug to exert its antitumor effects, it must not only penetrate the cell but also remain in its bioactive form to disrupt tumor growth [11]. Tumor cells evade therapy by activating enzymatic pathways that either inactivate the drug intracellularly or impair its conversion to an active metabolite [11]. The main enzymes responsible for this process are the cytochrome P450 (CYP) system, glutathione S-transferase (GST), and uridine diphospho-glucuronosyltransferase (UGT) [81]. The inactivation of drugs or the reduced activation by biochemical mechanisms of tumor cells is related to chemoresistance in both humans and animals. This highlights the need for comparative studies to identify conserved pathways and develop innovative therapeutic approaches [11].

### 3.5. Changes in the Drug Targets

Resistance mechanisms primarily driven by epigenetic changes or DNA mutations can influence the quantity or the structure of enzymes related to the cell cycle, resulting in modifications of cellular targets [11,35]. These changes may either amplify the activity of compounds that inhibit these targets or downregulate target expression, enabling cancer cells to evade chemotherapy [56]. Mutations and dysregulation of drug targets are thus critical determinants of therapeutic efficacy.

Topoisomerases regulate DNA topology during replication and transcription. Topoisomerase II facilitates DNA replication and transcription by creating transient double-strand breaks to relieve supercoiling [82]. Drugs like doxorubicin, etoposide, and mitoxantrone inhibit topoisomerase II; however, mutations in the enzyme can render these agents ineffective [29,32,56]. Similarly, topoisomerase I resolves single-strand DNA tension during replication. Camptothecin derivatives (e.g., irinotecan) inhibit topoisomerase I by stabilizing DNA–enzyme complexes, inducing lethal DNA damage. Mutations in topoisomerase I disrupt this interaction, allowing tumor cells to proliferate despite treatment [11,29].

In humans, mutations in the BCR-ABL fusion protein, commonly seen in chronic myeloid leukemia (CML), can confer resistance to tyrosine kinase inhibitors such as imatinib by altering the ATP-binding site of the kinase domain [83]. This requires the use of second- or third-generation inhibitors to overcome resistance [83]. Additionally, alterations in the estrogen receptor (ER) in hormone receptor-positive breast cancer reduce the efficacy of endocrine therapies like tamoxifen by inducing conformational changes in the ligand-binding domain that hinder the drug’s ability to antagonize receptor activity [84]. Such examples underscore how modifications in drug targets, whether through mutation or conformational changes, critically undermine therapeutic efficacy and pose significant challenges to long-term treatment success.

In veterinary medicine, alterations in drug targets have also been implicated in therapeutic resistance in both dogs and cats. In canine lymphoma, mutations in the ABCB1 gene may not only increase drug efflux but also impact the expression and conformation of intracellular drug targets, contributing to reduced sensitivity to chemotherapeutics like vincristine and doxorubicin [85]. In canine mast cell tumors, mutations in the c-KIT proto-oncogene can lead to constitutive activation of the KIT receptor, which is the therapeutic target of tyrosine kinase inhibitors such as toceranib and masitinib [86]. These mutations can alter the receptor’s structure and reduce drug-binding affinity, resulting in therapeutic failure [86]. Similarly, in feline injection-site sarcomas, overexpression or mutation of platelet-derived growth factor receptors (PDGFRs) may contribute to resistance to kinase inhibitors, although data are still emerging [87]. These cases demonstrate that, as in human oncology, mutations affecting drug targets in companion animals can significantly impact the success of targeted cancer therapies.

### 3.6. Apoptosis Inhibition

Apoptosis is a complex physiological mechanism that configures programmed cell death involving a cascade of energy-dependent molecular events divided into intrinsic and extrinsic pathways [88]. These pathways are interconnected, and molecules from one pathway can influence the other [89,90]. Triggered by extracellular ligands (e.g., TNF-α) binding to death receptors like tumor necrosis factor receptor (TNF-R), the extrinsic pathway activates procaspase-8, initiating a proteolytic cascade that cleaves structural proteins (e.g., actin and nuclear lamins), ultimately leading to the fragmentation of the genome and the cell [56,89,90]. The ultimate goal of this process is for the resulting apoptotic bodies to be efficiently engulfed by macrophages or other phagocytic cells, ensuring the orderly removal of cellular contents without inducing local inflammation. In the intrinsic pathway, a variety of non-receptor-mediated stimuli occur, which generate intracellular signals that act directly on targets within the cell, such as mitochondrial-initiated events by proteins such as Bcl2, AKT, Bax, and caspase-9 [56,89,90].

Chemotherapy damages DNA, subsequently resulting in cell death. However, changes in apoptosis or cell cycle signaling may cause resistance to chemotherapy in tumor cells via dysfunctional apoptosis [3,32]. The inhibition of apoptosis therefore leads to resistance to cell death and is characterized as a hallmark of cancer [4]. Studies in humans show the efficacy of anti-apoptotic B-cell lymphoma-2 (BCL-2) proteins, inhibitor-of-apoptosis proteins (IAPs), and cellular FLICE-inhibitory proteins (c-FLIPs) [51]. Mutations in and amplifications, chromosomal translocations, and overexpression of the genes encoding these previous proteins have been associated with various malignancies and linked to resistance to chemotherapy and targeted therapies [57].

Survivin, a caspase-inhibiting protein, blocks apoptosis by suppressing caspase activation. In canine lymphoma, survivin overexpression is linked to intrinsic (primary) resistance, as its immunoreactivity remains unchanged between initial tumors and relapses [91]. Similarly, survivin is upregulated in human carcinomas, sarcomas, and hematologic malignancies, correlating with poor therapeutic responses [91].

#### 3.6.1. The BCL-2 Family of Proteins

The BCL-2 family of proteins, characterized by evolutionarily conserved BCL-2 homology (BH) domains, plays a key role in regulating programmed cell death [92,93]. Typically, the anti-apoptotic and pro-apoptotic members of the BCL-2 family compete dynamically to influence the activation of pore-forming executors through interactions involving the BH3 domains [94,95].

Overexpression of anti-apoptotic proteins (BCL-2, BCL-xL, and MCL-1) has been linked to tumor initiation and progression [95,96]. In addition, it is observed that overexpression of BCL-2 confers resistance to cytotoxic chemotherapeutic agents in leukemic cells and thymocytes of mice [51]. Pro-apoptotic members (BAX and BAK) and BH3-only proteins (e.g., BID and BIM) counterbalance survival signals, modulating chemotherapy-induced apoptosis [97,98].

#### 3.6.2. Inhibitor-of-Apoptosis Proteins (IAPs)

The inhibitor-of-apoptosis protein (IAP) family is characterized by the presence of one to three IAP repeat domains (BIRs), which facilitate protein–protein interactions [94]. IAPs play several roles, including blocking apoptosis, modulating cell cycle progression, regulating receptor-mediated signal transduction, and activating the NF-κB signaling pathway [99]. These endogenous caspase inhibitors play an important role in preventing both apoptosis and a programmed form of necrosis called necroptosis [34,90]. In many cancers, like breast cancer, lung cancer, leukemias, and prostate cancer, abnormal high expression of IAPs has been linked to increased resistance to apoptotic stimuli [35,88,99]. This dysregulation is driven by oncogenic signals (e.g., Ras and HPV E6) and sustained NF-κB activity, which transcriptionally upregulate IAP expression [88].

#### 3.6.3. Cellular FLICE-Inhibitory Protein (C-FLIP)

The FLICE inhibitor cell protein (c-FLIP) is a critical suppressor of apoptosis mediated by death receptors, such as Fas, TNF-R1, DR4, and DR5. Its main function is to block the activation of the apoptotic cascade, preventing the dimerization and activation of procaspase-8 through the recruitment of c-FLIP to the death-inducing signaling complex (DISC) or to complex II [88,100,101]. At high levels of expression, the three isoforms of c-FLIP act by inhibiting the activation of procaspase-8 and procaspase-10 [88,102]. Overexpression of c-FLIP isoforms is observed in colorectal carcinoma, chronic B-cell lymphocytic leukemia, and other malignancies, correlating with poor treatment responses [100,101]. Beyond death receptor signaling, c-FLIP also disrupts apoptosis induced by chemotherapeutic agents. Its overexpression is implicated in drug resistance by dysregulating apoptotic and cell cycle signaling pathways [57].

### 3.7. Multidrug Resistance

Multidrug resistance (MDR) in tumors can be conceptualized through two distinct mechanisms. The first is through the sum of several acquired resistance mechanisms. For example, cisplatin resistance involves altered drug influx, enhanced DNA repair, and other compensatory mechanisms [17]. The second refers to multidrug resistance resulting from changes in one or more ABC transporters, which increase drug efflux, thus reducing intracellular chemotherapeutic concentrations [17,18,103]. Additional contributors to MDR include microRNAs, hypoxia induction, and epigenetic regulation [103].

ABC transporters are cell membrane-bound proteins that control the efflux of compounds from the intracellular to extracellular space [33]. Their overexpression compromises chemotherapy by depleting intracellular drug levels. In canine lymphoma, MDR1 may be overexpressed, reducing the concentration gradient of doxorubicin and vincristine—cornerstones of lymphoma treatment—by reducing their intracellular retention [33] Table 1 summarizes key chemoresistance mechanisms related to anticancer drugs.

## 4. Conclusions

This review highlights the complex and multifactorial nature of chemotherapy resistance in both human and veterinary oncology. The mechanisms described—such as altered drug influx and efflux, enhanced DNA repair, metabolic reprogramming, target modifications, and inhibition of apoptosis—are consistent with findings reported in previous human oncology studies. These conserved pathways suggest that cancer in companion animals shares fundamental resistance traits with human tumors, reinforcing the value of comparative oncology as a tool to accelerate understanding and innovation. In summary, chemoresistance in dogs and cats presents a significant challenge in veterinary oncology, limiting the effectiveness of treatments and reducing long-term remission rates. While chemotherapy resistance is well-documented in human cancers, research in companion animals remains limited—particularly in cats, where mechanistic studies are nearly absent. Bridging this gap requires a comparative oncology framework, leveraging insights from human studies to accelerate discoveries in veterinary medicine. To enhance the understanding of cancer, the use of data science and large-scale data analysis will be essential for grasping its complexity on both cellular and population scales. Future research should prioritize large-scale genomic and transcriptomic studies in veterinary patients to better characterize resistance markers and identify actionable targets. The integration of big data, bioinformatics, and molecular biology within a One Health framework may drive the development of predictive models and novel therapies capable of overcoming chemotherapy resistance. Ultimately, a deeper understanding of these mechanisms will enhance therapeutic success and improve outcomes for both human and companion animal cancer patients, thereby enhancing their quality of life.

## Figures and Tables

**Table 1:** Summary of chemoresistance mechanisms and associated cancer types.

Resistance Mechanism	Chemotherapeutic Agents	Cancer Type	References
Increased output and decreased input	Vinca alkaloids (vinblastine, vincristine, and catharanthine), anthracyclines (doxorubicin and daunorubicin), taxanes (paclitaxel and docetaxel), epipodophyllotoxins (etoposide and teniposide), camptothecins derivates (topotecan and methotrexate), anthracenes (bisantrene and mitoxantrone), platinum compounds (for example, cisplatin and oxaliplatin), and proteolysis-targeting chimera drugs.	Canine lymphoma; acute lymphoblastic leukemia, non-small-cell lung carcinoma (NSCLC), human ovarian cancer, canine mammary tumor cell, acute myeloid leukemia, human colon carcinoma, human breast carcinoma, human gastric carcinoma, human fibrosarcoma, human glioblastoma, human myeloma, human prostate cancer cell, and human osteosarcoma.	[31,32,35,36,44,45,46,48,49,50,51,70,104]
Enhanced DNA repair	Vinca alkaloids (vinblastine, vincristine, and catharanthine), platinum compounds (cisplatin and oxaliplatin), anthracyclines (doxorubicin), and topoisomerase I inhibitors (irinotecan).	Human ovarian cancer, human testicular cancer, sarcoma, canine lymphoma, human osteosarcoma, and human small-cell lung carcinoma.	[16,32,35,56,70,105]
Metabolic changes and detoxification	Antimetabolites (5-fluorouracil, cytosine arabinoside, methotrexate, gemcitabine, and cytarabine), vinca alkaloids (vinblastine, vincristine, and catharanthine), and platinum compounds (cisplatin and oxaliplatin).	Human breast cancer, human colorectal cancer, human pancreatic cancer, human gastric cancer, human head and neck cancer, ovarian cancer, canine lymphoma, leukemia, human testicular cancer, human osteosarcoma, and human small-cell lung carcinoma.	[16,32,56,65,106,107]
Tumor microenvironment modulation	mTORC1 inhibitors, PI3K, platinum drugs, DOX, VEGFR1/2/3 inhibitors, histone deacetylase inhibitors, phosphatidylinositol3-kinase, anti-VEF drugs, and taxane drugs.	Human esophageal cancer, human prostate cancer, and human breast cancer.	[108,109,110,111,112,113]
Cell-targets modification	Camptothecins derivatives (topotecan and methotrexate) and topoisomerase I inhibitors (irinotecan).	Human breast cancer, canine lymphoma, human leukemia, human colorectal cancer, human small-cell lung carcinoma and human chronic myelogenous leukemia, and human osteosarcoma.	[16,32,35,56,104,114]
Apoptosis inhibition	Vinca alkaloids (vinblastine, vincristine, and catharanthine), platinum compounds (cisplatin), anthracyclines (doxorubicin), epipodophyllotoxins (etoposide), and topoisomerase I inhibitors (irinotecan).	Human colorectal cancer, human small-cell lung carcinoma, human gastric adenocarcinoma, human anaplastic thyroid cancer, human pancreatic carcinoma, human Kaposi’s sarcoma, human Ewing’s sarcoma, human non-small-cell lung cancer, human breast cancer, canine lymphoma, human leukemia, human myeloma, human osteosarcoma, human glioblastoma, human melanoma, feline mammary carcinoma, canine osteosarcoma, and mast cell tumor.	[29,32,35,50,65,88,95,99,101,111,115,116,117,118,119]

## Data Availability

All data can be retrieved from electronic databases (PubMed, Google Scholar, Scopus, Embase, and Cochrane).

## Abbreviations

The following abbreviations are used in this manuscript:

ABC	ATP-binding cassette
BCL-2	B cell lymphoma-2
BCRP	Breast cancer resistance protein
c-FLIP	Cellular FLICE-inhibitory protein
CML	Chronic myeloid leukemia
CYP	Cytochrome P450
DCK	Deoxycytidine kinase
DISC	Death-inducing signaling complex
DOX	Doxorubicin
ER	Estrogen receptor
FPGS	Folylpolyglutamate synthetase
GGH	γ-glutamyl-hydrolase
GLUT2	Glucose transporter 2
GST	Glutathione S-transferases
HR	Homologous recombination
IAPs	Inhibitor-of-apoptosis proteins
MDR1	Multidrug resistance protein 1
MRP1	MDR-associated protein 1
MRR	Homologous recombination repair
NER	Nucleotide excision repair system
NSCLC	Non-small-cell lung carcinoma
PDGFR	Platelet-derived growth factor receptors
PgP	P-glycoprotein
RFC-1	Reduced folate carrier 1
SLC	Solute Carrier Family
TLA	Three-letter acronym
TS	Thymidylate synthase
TYMS	Thymidylate synthase gene
UGT	Uridine diphospho-glucuronosyltransferase
VEGFR1/2/3	Vascular endothelial growth factor receptors 1, 2, and 3
